# Different Alterations in Gut Microbiota between *Bifidobacterium longum* and Fecal Microbiota Transplantation Treatments in Propionic Acid Rat Model of Autism

**DOI:** 10.3390/nu14030608

**Published:** 2022-01-30

**Authors:** Turki S. Abujamel, Norah M. Al-Otaibi, Sameera Abuaish, Rahaf H. AlHarbi, Mushref B. Assas, Saleha Ahmad Alzahrani, Sohailah Masoud Alotaibi, Afaf El-Ansary, Kawther Aabed

**Affiliations:** 1 Vaccines and Immunotherapy Unit, King Fahd Medical Research Center, King Abdulaziz University, Jeddah 21589, Saudi Arabia; rahafh_h@live.com; 2 Department of Medical Laboratory Sciences, Faculty of Applied Medical Sciences, King Abdulaziz University, Jeddah 21589, Saudi Arabia; massas@kau.edu.sa; 3 Department of Biology, College of Science, Princess Nourah Bint Abdulrahman University, P.O. Box 84428, Riyadh 11671, Saudi Arabia; noramajd22@gmail.com (N.M.A.-O.); salehah1416@gmail.com (S.A.A.); sohailah1997@gmail.com (S.M.A.); dr.kaabed@gmail.com (K.A.); 4 Department of Basic Sciences, College of Medicine, Princess Nourah Bint Abdulrahman University, P.O. Box 84428, Riyadh 11671, Saudi Arabia; syabuaish@pnu.edu.sa; 5 Central Laboratory, Female Center for Medical Studies and Scientific Section, King Saud University, P.O. Box 22452, Riyadh 11472, Saudi Arabia; elansary@ksu.edu.sa

**Keywords:** autistic spectrum disorders, probiotics, *Bifidobacterium longum*, microbiota, fecal microbiota transplantation

## Abstract

Autism spectrum disorders (ASD) consist of a range of neurodevelopmental conditions accompanied by dysbiosis of gut microbiota. Therefore, a number of microbiota manipulation strategies were developed to restore their balance. However, a comprehensive comparison of the various methods on gut microbiota is still lacking. Here, we evaluated the effect of *Bifidobacterium* (BF) treatment and fecal microbiota transplantation (FT) on gut microbiota in a propionic acid (PPA) rat model of autism using 16S rRNA sequencing. Following PPA treatment, gut microbiota showed depletion of Bacteroidia and *Akkermansia* accompanied by a concomitant increase of *Streptococcus*, *Lachnospiraceae*, and *Paraeggerthella*. The dysbiosis was predicted to cause increased levels of porphyrin metabolism and impairments of acyl-CoA thioesterase and ubiquinone biosynthesis. On the contrary, BF and FT treatments resulted in a distinct increase of *Clostridium*, *Bifidobacterium*, *Marvinbryantia*, *Butyricicoccus*, and *Dorea*. The taxa in BF group positively correlated with vitamin B12 and flagella biosynthesis, while FT mainly enriched flagella biosynthesis. In contrast, BF and FT treatments negatively correlated with succinate biosynthesis, pyruvate metabolism, nitrogen metabolism, beta-Lactam resistance, and peptidoglycan biosynthesis. Therefore, the present study demonstrated that BF and FT treatments restored the PPA-induced dysbiosis in a treatment-specific manner.

## 1. Introduction

Autism spectrum disorders (ASD) are a group of conditions categorized by a variety of neurodevelopmental and behavioral malfunctions that have come to form archetypal features related to the disorder [1]. The global prevalence of ASD is estimated to be as high as 0.7%; however, this percentage is increasing markedly with more invasive and multi-layered studies on larger groups with less variables and more scrutinizing criteria [2,3]. Furthermore, and along with well-characterized social deficits that burden autistic individuals from all spectrums within the disorder, pervasive and ill-understood systemic inflammation, gastrointestinal tract-(GIT) related problems corroborating with changes in the gastrointestinal microbiota composition with links to the neuroimmune-gut brain axis are considered characteristic of people with autism [4].

Recent studies have suggested a relationship between gut microbiota and the commencement of emotional and behavioural traits indicating a relatively strong gut-brain axis link [5]. Additionally, Y Wang et al. [6] listed neuropsychiatric and neuroimmune disorders associated with variations in microbiome populations. Diseases like depression, anxiety and ASD were all associated with different patterns of variations in microbiota, indicating a link between these diseases and gut bacteria [6]. In fact, gut microbiota has been associated with a range of neuro-related diseases, i.e., neuromyelitis optica, Guillain-Barré syndrome (GBS), and multiple sclerosis. In multiple sclerosis, treatment with the probiotic *Bifidobacterium* improved symptoms in experimental autoimmune encephalitis rat models [7]. Neuromyelitis patients had antibodies that cross react with *Clostridium perfringens* proteins [8] and cross reactivity between antibodies against *campylobacter jejuni*, the common cause agents for human enteritis, and the neural surface antigens is understood to be a key the risk factor for GBS [9].

The gastrointestinal tract is the largest surface in the body with tremendous numbers of different microorganisms that outnumber human cells. These microorganisms interact together within the gut’s ecosystem while dictating the host’s many vital-life functions, i.e., nutrition and immunity [10,11]. It is wildly accepted that a healthy gut sustains a ‘healthy’ microbial composition. This was further consumed as increasing evidence suggested concurrent links between microbial dysbiosis, which is defined as a deviation from the normal balance of microbiota, and a range of gut-related diseases such as inflammatory bowel disease and irritable bowel syndrome [11,12]. Dysbiosis was a common feature not only in gut-related diseases but within a range of other illnesses that target sites not associated with the GIT in the body especially those relating to the nervous system and muscular-motor function disorders such as Parkinson’s disease, multiple sclerosis, and most recently ASD [13]. Indeed, abnormalities in microbial populations within the gut is found repeatedly in ASD patients [14,15].

Dysbiosis of gut microbiota was demonstrated in several animal models of autism and human studies. These studies suggested the preference of the gut’s environment for abnormal bacterial species in ASD. Abnormal Firmicutes to Bacteroidetes have been reported in biopsy samples from ASD children [16,17,18]. These findings were attributed to the compositional dysbiosis with the imbalance between the two bacterial families varying throughout the different compartments of the gut ranging from (while not limited to) the illume to as far down to the rectum [19]. ASD patients consistently show a dysfunctional imbalance by displaying elevated levels of Proteobacteria [20], *Lactobacillus* [21], *Bacteroides* [22], *Desulfovibrio* [23], and *Clostridium* [24]. This is usually accompanied by decreased levels of *Bifidobacterium* [25], *Blautia* [24], *Dialister* [26], *Prevotella* [27], *Veillonella*, and *Turicibacter* [26], all in stark contrast to the bacterial profile in healthy individuals. It is worth noting that *Bifidobacterium* in particular has been found to be repeatedly decreased in ASD children [16,17,28]. Importantly, *Bifidobacterium* is one of the most abundant members of the gut microbiota, especially in breast-fed infants, and is widely considered beneficial for the host’s gut health. For instance, gamma-aminobutyric acid (GABA) is a pivotal neurotransmitter with inhibitory functions modulating nerve activation and voltage conductions [29]. GABA levels have been reported to be altered in ASD children in comparison to control groups [27], and this has been attributed to the decrease in *Bifidobacterium* spp. that is known to be key producers of GABA in the gut [30]. Clinical trials using a diverse range of antibiotics in early attempts to manipulate the bacterial communities within the gut helped us understand and asses the behavioral impact of anti-microbial therapy in treated individuals [16,31]. Indeed, combination therapy with vancomycin and *Bifidobacterium* improved over all autistic symptoms as assessed by the “Autism Behavior Checklist” and stabilized the levels of 3-(3-hydroxyphenyl)-3-hydroxypropionic acid, 3-hydroxyphenylacetic acid, and 3-hydroxyhippuric acid in urine samples of ASD patients [32].

Attempts to restore bacterial healthy compositions in the gut, while controversial at times, has seen bacterial reconstitution being able to alter the course of behavioral disorders in ASD murine models [33]. Another contentious model of microbiota alteration, fecal microbiota transfer (FT) therapy, has gained attention in recent years [34,35]. FT improved GIT impairments and neurobehavioral symptoms in ASD patients [25]. Indeed, FT was successful in reliving gut hypersensitivity and inflammation. Moreover, prebiotics, probiotics such as *Bifidobacterium*, and FT are increasingly used to neutralize any colonization of harmful bacteria while aiding nutritional and balance immune functions.

In this study, we discuss the different microbiome alteration in an induced autism rat model. We compare between the two commonly practiced models of microbiome modulation, *Bifidobacterium longum*, and FT administrations, and investigate the different bacterial correlation in response.

## 2. Materials and Methods

### 2.1. Animal Experiment

**Figure 1:**
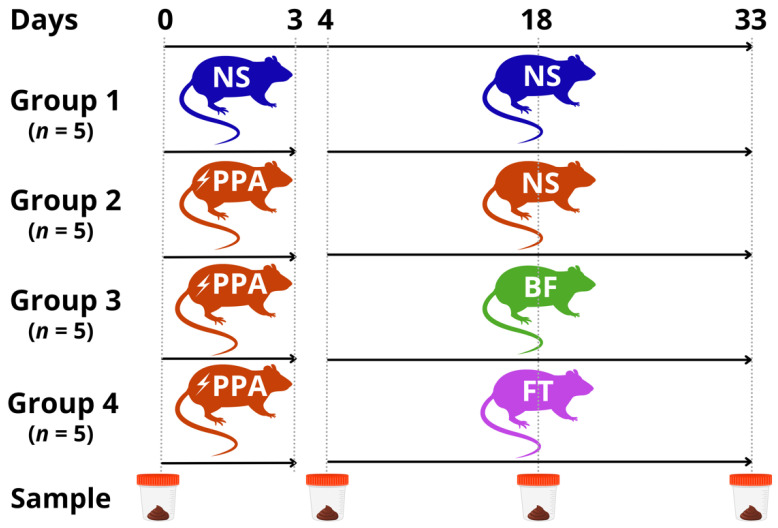
Schematic representation of the experimental design. Normal Saline (NS); propionic acid (PPA); *Bifidobacterium longum* (BF); or fecal microbiota transplantation (FT).

Twenty male Sprague Dawley rats (28 days old), with weight ranging from 80–120 g, were used in the study. The animals were housed in a controlled environment with 12 h light/dark cycle and temperature of 22 °C ± 2 °C. Both food and water were provided ad libitum. The animals were divided into four groups (*n* = 5 each), in which the control group was given 1 mL of normal saline (NS) daily for 3 days, followed by daily dose of 1 mL NS from day 4 to 33. The remaining groups were given a daily dose of 250 mg/kg of propionic acid (PPA) for 3 days to induce neurotoxicity [36]. On the fourth day, the animals received either 1 mL NS daily (PPA group), 1 × 10^9^ colony forming units (CFU) *Bifidobacteria* (BF group), or 1 g/kg fecal transplant (FT group) for the remaining 30 days. The *Bifidobacteria* suspension was prepared by dissolving one *Bifidobacterium longum* BB536 capsule containing 25 mg of 2 × 10^9^ CFU (Bifido GI balance, Life Extension) in 1 mL sterile phosphate-buffered saline (PBS, pH 7.4; Gibco^TM^). Of that suspension, 0.5 mL was used for oral gavage [37]. On the other hand, the fecal transplantation suspension was prepared by homogenizing 1 g of freshly voided feces from healthy donor rats in 10 mL of sterile PBS. The homogenate was then filtered through a sterilized metal sieve, then the final filtrate was used for oral gavage at 1 g/kg [38]. Fresh stool samples were collected from each animal in a sterile microcentrifuge tube on days 0 (before the start of treatment), day 4 (6 h following the second treatment, immediate effect), day 18 (14 days of the second treatment, intermediate effect), and day 33 (30 days of the second treatment, long-term effect) (Figure 1). Next, the stool samples were stored at −80 °C until processed for nucleic acid extraction.

### 2.2. Social Behavior Evaluation

The alteration in animal neurological behavior was evaluated utilizing the three-chamber social test as described previously [34,39,40]. Briefly, each animal was individually placed in the center of a three-chamber box and was allowed to explore for 5 min with the doorways closed. Next, the two doors were opened and a new rat with the same sex and body weight was placed in one of the perforated holding containers and was allowed to explore the different chambers for 10 min. Each trial was recorded with HD camcorder (Legria, Canon). The recorded videos were then analyzed using BORIS 7.9.16 software [41]. The parameters investigated included the time of social interaction and the time spent in the different areas of the chambers.

### 2.3. Metagenomic DNA Extraction and Next-Generation Sequencing

DNA was extracted from the fecal samples using the QIAamp DNA soil Kit (Qiagen, Hilden, Germany), following the manufacturer’s instructions. Then, the concentration and purity of the extracted DNA was measured using a NanoDrop spectrophotometer (Thermo Fisher Scientific Inc., Waltham, MA, USA). Next, one-hundred ng of the extracted DNA from each sample was shipped to Macrogen Inc. (Seoul, Korea) for 16S rRNA paired-end sequencing using Illumina MiSeq platform with Reagent Kit v3 (Illumina Inc., San Diego, CA, USA), targeting the V3–V4 region.

### 2.4. Bioinformatics Analysis

The demultiplexed paired-end sequencing data were analyzed using the Quantitative Insights into Microbial Ecology 2 (QIIME 2) pipeline V2021.2 [42]. Briefly, the sequences were quality filtered and denoised using the DADA2 package [43] with forward and reverse truncation set to 250 bp. Then, the denoised reads were mapped to the 97% Operational Taxonomic Units (OTU) representative sequences of the Greengenes database V13_8 [44] with 95% minimum identity threshold utilizing the Vsearch tool [45]. OTUs with frequency <5 reads were filtered out from subsequent analysis. Next, alpha diversity (represented by Chao1 and Shannon diversity indices) and beta diversity using unweighted and weighted UniFrac distances [46] were computed in QIIME2 using 24,000 reads per samples, which represents the minimum number of reads observed in an individual sample. Finally, the abundance of the identified taxa was summarized in bar plots at the phylum level using QIIME2, and the abundance at the genus level was presented in heatmap using Morpheus tool (https://software.broadinstitute.org/morpheus) (accessed on 13 October 2021). Subsequently, the differentially abundant taxa between different groups were identified using the Linear discriminant analysis Effect Size (LEfSe) tool [47]. In brief, the OTUs frequency table was uploaded on the Galaxy Version 1.0 online interface (https://huttenhower.sph.harvard.edu/galaxy/) (accessed on 5 November 2021). Then, the LDA score was computed, setting the Wilcoxon *p*-value at 0.05 and the logarithmic LDA score cutoff to 2.0. Spearman correlation was calculated to show the association between the identified taxa and timepoints for each experimental condition using the Calypso tool V8.84 [48]. Additionally, the Calypso tool was used to determine the microbiota co-occurrence network at the genus level using taxon-taxon Bray–Curtis dissimilarities and Spearman correlation. The *p*-values for the correlation analysis were combined using Simes association with 0.05 FDR cutoff. Subsequently, the metagenome of the identified microbiota was predicted using the Phylogenetic Investigation of Communities by Reconstruction of Unobserved States 2 (PICRUSt2) pipeline [49] integrated in QIIME2, utilizing the pcirust2_pipeline.py command and using the denoised and filtered representative sequences as input, aligning the representative sequences against the Kyoto Encyclopedia of Genes and Genomes (KEGG) database [50]. Next, the annotation of the predicted genes was done using the add_descriptions.py command. The predicted genes were clustered into pathways, and a correlation between the identified genes and the duration of treatment in each condition was calculated using Spearman correlation utilizing the MicrobiomeAnalyst tool [51,52].

### 2.5. Statistical Analysis

Alpha diversities were compared between the groups at each time point, or within the same group at different time-points, using Kruskal–Wallis H test followed by Dunn’s multiple comparison test utilizing GraphPad Prism V9.0 (GraphPad Software, San Diego, CA, USA), and *p* ≤ 0.05 was considered significant. The nonparametric analysis of similarities (ANOSIM) [53] was used to determine the significance in unweighted and weighted UniFrac distances within and between groups utilizing QIIME2 with 999 permutations. LEfSe [47] was used to identify the differentially abundant taxa. Moreover, the correlation between KEGG orthologs within and between groups were calculated using Spearman correlation analysis integrated in the MicrobiomeAnalyst tool [51,52], with FDR corrected *p*-values ≤ 0.05 considered significant.

## 3. Results

### 3.1. Social Behavior Impairment and Rescue

In our recent publication [34], we illustrated that rats treated with PPA showed impaired social behavior exhibited by reduced time spent in the social chamber and time interacting with the social stimulus compared to NS control group. Both BF and FT treatments restored this to control levels. Moreover, PPA treated rats showed increased immobility, with both BF and FT treatments restoring rat behavior [34].

### 3.2. Microbiota Diversity Is Enriched upon BF and FT Treatments

**Figure 2:**
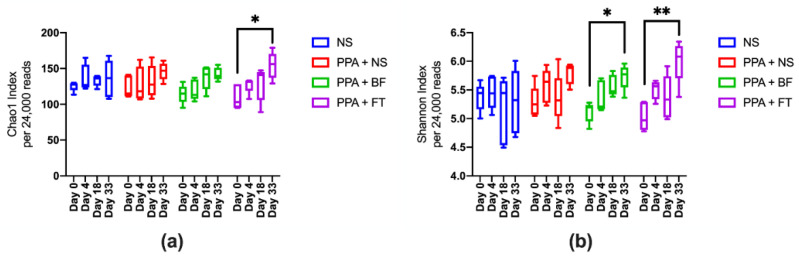
Richness (Chao1) and diversity (Shannon) indices of gut microbiota along the treatment duration. (**a**) Chao1 and (**b**) and Shannon indices of gut microbiota of each treatment group was compared between the different timepoints. Total number of reads per sample was equalized to 24,000 reads. NS, normal saline group; PPA + NS, propionic acid control group; PPA + BF, *Bifidobacterium* treatment group; PPA + FT, fecal microbiota transplantation treatment. * *p* ≤ 0.05, ** *p* ≤ 0.001.

**Figure 3:**
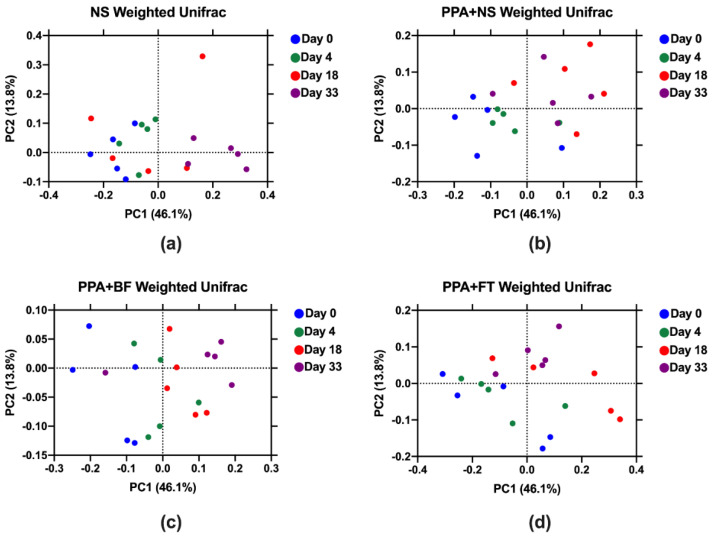
Principal coordinate analysis of weighted UniFrac distances in each group. Total number of reads per samples was equalized to 24,000. Normal saline (NS) control group (**a**), propionic acid (PPA + NS) control group (**b**), *Bifidobacterium* (PPA + BF) treated group (**c**), and fecal microbiota transplantation treated (PPA + FT) group (**d**). Each circle represents one sample, and percentage of variance explained by each component is presented for each axis.

We evaluated the diversity of fecal microbiota between different groups through analyzing the V3–V4 hypervariable region of the 16S rRNA gene using next-generation sequencing. The sequencing resulted in a total of 2,562,975 high-quality paired-end reads, with an average of 32,037 ± 2954 reads per sample, ranging from 24,347 to 40,281 high-quality reads per sample (Table S1). Comparing the alpha-diversity (Chao1 and Shannon diversity indices), which describes the microbiota diversity within samples, between the different interventions at each timepoint showed no significant difference (*p* > 0.05) (Figure S1). However, a gradual increase in the microbiota diversity within the BF and FT treatment groups correlated with the duration of the treatment. This displayed a significant increase in Chao1 index in day 33 compared to day 0 in the FT treatment group (*p* = 0.012), and similar significant increase was observed in Shannon diversity index in the BF (*p* = 0.012) and FT (*p* = 0.005) treatment groups (Figure 2). Principal component analysis (PCA) showed a better separation of samples belonging to the same timepoints within each treatment group in both unweighted (analysis of similarities (ANOSIM) R^2^ = 0.25; *p* = 0.001) (Figure S2) and weighted UniFrac distances (ANOSIM R^2^ = 0.28; *p* = 0.001) (Figure 3) compared to that between groups (unweighted distances ANOSIM R^2^ = 0.04; *p* = 0.028, and weighted UniFrac distances ANOSIM R^2^ = 0.006; *p* = 0.34) (Figure S3).

### 3.3. Microbiota Composition Alteration Was Different between Treatments

**Figure 4:**
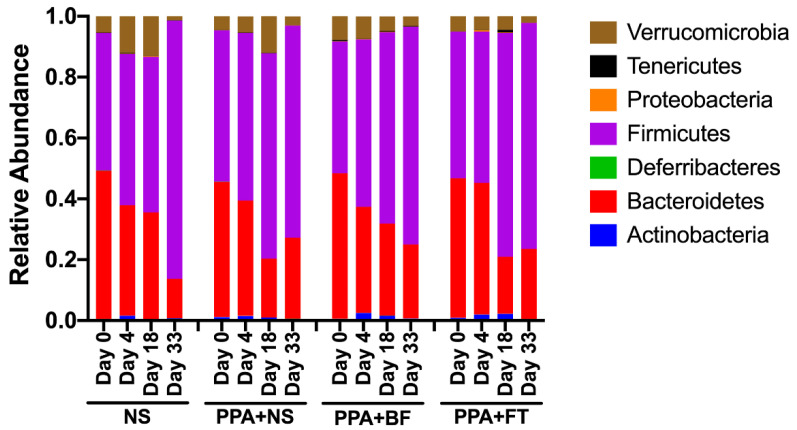
Identified bacterial taxa at the phylum level. Relative abundance of assigned bacterial phyla in all samples are clustered at each experimental group. Each column represents the mean relative abundance of the identified phyla at each timepoint per group.

**Figure 5:**
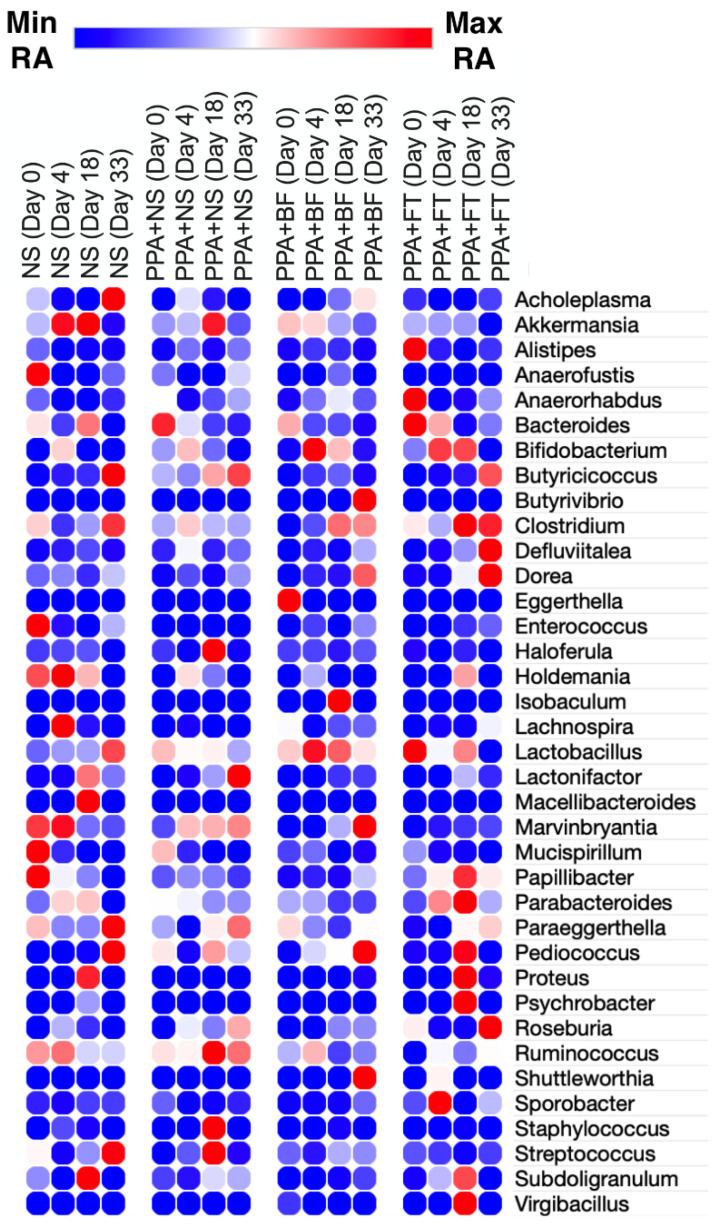
Identified genera clustered by intervention group. Each circle represents the mean relative abundance of the identified genus from animals within a treatment group at each timepoint (*n* = 5). NS, Normal saline control group; PPA + NS; propionic acid control group; PPA + BF, *Bifidobacterium* treated group; PPA + FT, fecal microbiota transplantation treated group. Scale represents a color scheme for the minimum and maximum relative abundance (RA) values in each row.

The assigned bacterial taxa belonged to seven phyla (Table S2), arranged from the highest abundance to the lowest as follows: Firmicutes (59.42% ± 15.98), Bacteroidetes (33.26% ± 14.52), Verrucomicrobia (5.85% ± 6.90), Tenericutes (0.18% ± 0.5), Actinobacteria (1.14% ± 1.27), Proteobacteria (0.11% ± 0.2), and Deferribacteres (0.03% ± 0.09) (Figure 4, Table S3). The abundance of the identified bacterial phyla was not statistically significant (*p* > 0.05) when compared between and within groups. Within these phyla, a total of 37 bacterial genera were identified, and the top 10 most predominant genera in all groups were *Lactobacillus* (13.58% ± 8.62), *Clostridium* (11.79% ± 6.89), *Bacteroides* (7.10% ± 5.37), *Akkermansia* (5.80% ± 6.85), *Ruminococcus* (5.35% ± 4.64), *Butyricicoccus* (1.10% ± 1.56), *Bifidobacterium* (0.89% ± 1.32), *Lactonifactor* (0.81% ± 2.07), *Parabacteroides* (0.72% ± 0.83), and *Dorea* (0.51% ± 0.69) (Figure 5, Figures S4 and S5, Table S4).

**Figure 6:**
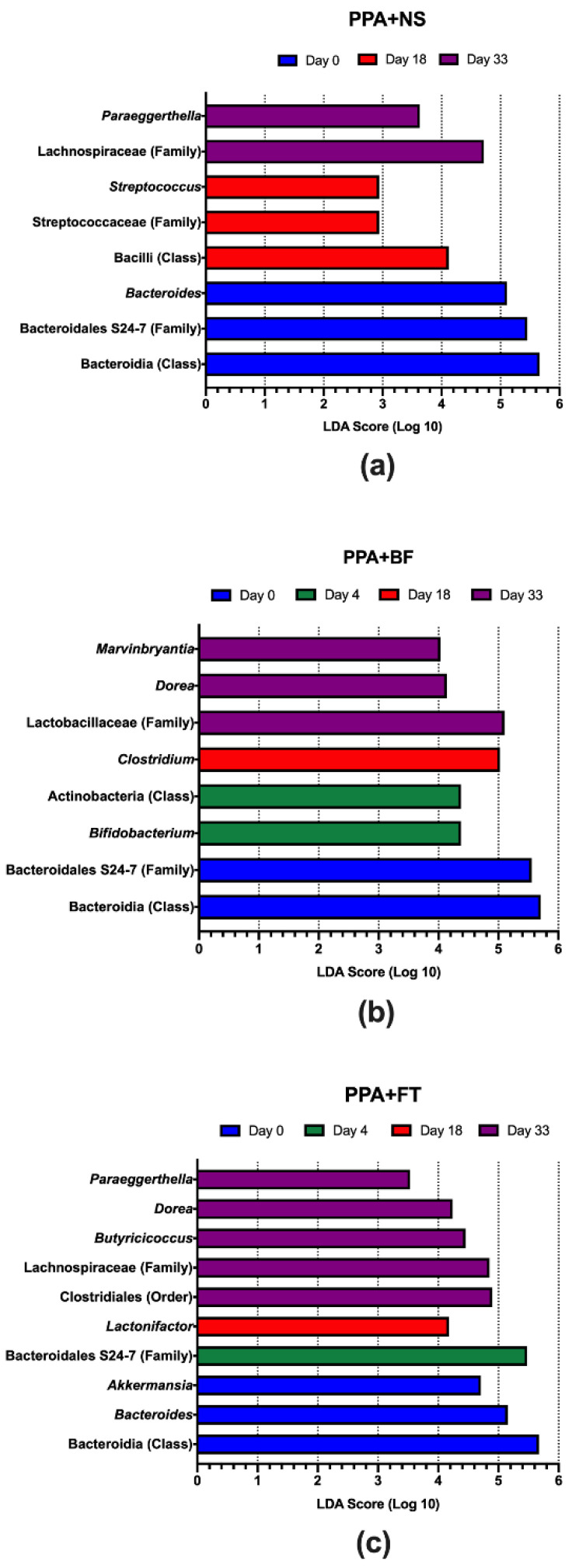
Linear discriminant analysis (LDA) effect size (LEfSe) of gut microbiota in different treatments. Relative abundance of gut microbiota was compared within the propionic acid (PPA + NS) group (**a**), the *Bifidobacterium* (PPA + BF) treatment group (**b**), and fecal microbiota transplantation (PPA + FT) treatment group (**c**). Only taxa with statistically significant differences (*p* ≤ 0.05) are presented. NS, normal saline.

Comparing the microbiota between the different timepoints in the PPA control group showed a significantly higher abundance of Bacteroidia (mainly represented by the family Bacteroidales S24-7 and *Bacteroides*) at day 0 (Figure 6a), which indicate a decrease upon PPA treatment. At day 18 (two weeks post PPA treatment), a significant increase was observed in Bacilli and Streptococcaceae (mainly represented by *Streptococcus*). At day 33 (30 days post PPA treatment), a significant enrichment in *Paraeggerthella* was observed. Similar to the PPA control group, the BF and FT treated groups showed significantly higher abundance of Bacteroidia (Figure 6b,c). Within BF treated group, there was a significant increase in Actinobacteria (mainly represented by *Bifidobacterium*) at day 4, i.e., 6 h after the first oral inoculation with *Bifidobacterium*, followed by a significant increase of *Clostridium* at day 18 (two weeks of BF treatment). At day 33 (30 days of BF treatment), there was a significant increase in Lactobacillaceae, *Dorea*, and *Marvinbryantia* (Figure 6b). On the other hand, the FT treated group showed a significant increase in *Akkermansia*, in addition to Bacteroidia, on day 0 (Figure 6c). Moreover, Bacteroidales S24-7 was enriched at day 4, followed by Lactonifactor at day 18. The terminal timepoint displayed a significant increase in Clostridiales, Lachnospiraceae, *Butyricicoccus*, *Dorea*, and *Paraeggerthella* (Figure 6c). Altogether, an increase or depletion of certain bacterial taxa was observed during each treatment group. We also compared the relative abundance of the identified taxa between groups at each timepoint to identify differentially abundant taxa. For instance, day 4 revealed a significant increase in the relative abundance of Bacillaceae in the FT treated group and in *Staphylococcus* in the NS control group (Figure S6a). At day 18, there was a significant enrichment in the Clostridiaceae family within the FT treated group and the Streptococcaceae family (mainly represented by *Streptococcus*) in the PPA control group (Figure S6b). At the terminal timepoint (day 33), there was a significant enrichment in the *Mollicutes*, *Parabacteroides*, and *Clostridium* in the FT treated group (Figure S6c).

**Figure 7:**
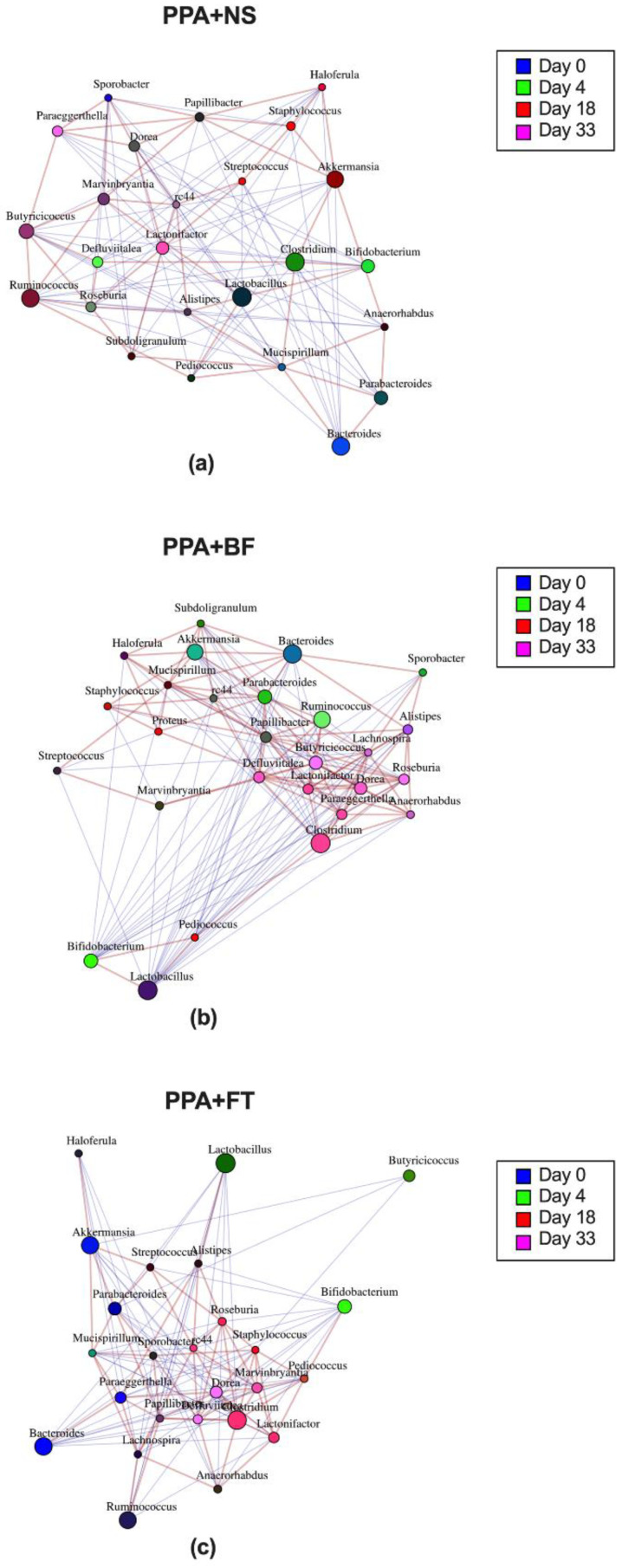
Spearman correlation co-occurrence network showing the 25 most abundant genera. The size of the circle is proportional to the bacterial relative abundance, and the red and blue lines represent positive and negative correlation, respectively. Circle colors denote the day of sample collection. Propionic acid (PPA + NS) control group (**a**), *Bifidobacterium* (PPA + BF) treated group (**b**), fecal microbiota transplantation treated (PPA + FT) group (**c**).

To determine the relationship between the identified bacterial genera during the intervention period, co-occurrence networks were constructed. In the PPA control group, there was a positive correlation between *Akkermansia* at days 18–33 with *Staphylococcus* and *Haloferula* at day 18 and Clostridium at day 4. This correlation was coupled with negative correlation with *Ruminococcus*, *Butyricicoccus*, *Defluviitalea*, *Roseburia*, and *Pediococcus*, mainly at day 4 and day 18 (Figure 7a). The BF treated group showed a positive correlation between *Bifidobacterium* at day 4 with *Pediococcus* on day 18. On the contrary, the increased abundance of *Bifidobacterium* was negatively correlated with *Alistipes* and *Streptococcus*, *Marvinbryantia*, *Anaerorhabdus*, and *Lachnospira* on day 18 and day 33. This group of bacteria, directly or indirectly, positively correlated with higher abundance of *Akkermansia* (Figure 7b). In the FT treated group, there was a positive correlation between *Bifidobacterium* on day 4, *Lactobacillus* on day 18 and day 33, and *Pedociccus* on day 18. The later genus had a positive correlation with *Clostridium*, which positively correlated with *Dorea* and *Roseburia* mainly on day 33. These two genera negatively correlated with several other taxa including *Akkermansia*, *Bacteroids*, *Haloferula*, *Mucispirillum*, and *Streptococcus* (Figure 7c).

To identify possible trends in bacterial abundance, Spearman correlation analysis between the relative abundance of bacterial genera along the treatment period was done. Spearman correlation identified 22 genera with significant correlation in all groups (Table S5). The top 5 genera with the highest correlation were *Akkermansia*, *Bifidobacterium*, *Butyricicoccus*, *Clostridium*, and *Dorea*. Treatment with BF and FT showed a negative correlation with *Akkermansia* (BF: R = −0.55, *p* = 012; FT: R = −0.65, *p* = 0.0021) throughout the study period (Figure S7). On the contrary, the PPA control group showed a negative correlation with *Bifidobacterium* (R = −0.45, *p* = 0.046). Furthermore, a strong positive correlation was observed with *Butyricoccus* in the NS control (R = 0.6, *p* = 0.005) and FT treated groups (R = 0.73, *p* = 0.0003). In addition, *Clostridium* had a negative correlation with the NS (R = −0.54, *p* = 0.014) and PPA control groups (R = −0.48, *p* = 0.033). Finally, *Dorea* showed a positive correlation following BF (R = 0.67, *p* = 0.0013) and FT treatments (R = 0.7, *p* = 0.0006) (Figure S7).

### 3.4. The Predicted Metagenome of the Identified Taxa Highlights the Importance of Key Metabolic Pathways in Each Treatment

**Figure 8:**
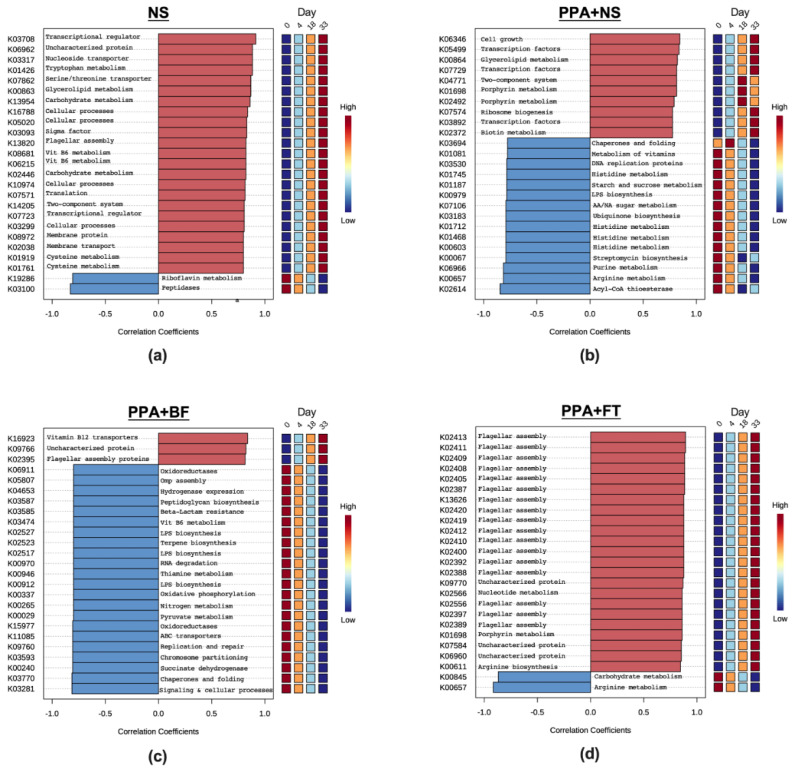
Correlation between the predicted KEGG Orthology annotations and intervention duration. Histograms show Spearman rank correlation coefficients (R) of the top 25 KEGG Orthology annotations with significant correlations (*p* < 0.05). Normal saline control (NS) group (**a**), propionic acid control (PPA + NS) group (**b**), *Bifidobacterium* treated (PPA + BF) group, (**c**), fecal microbiota transplantation treated (PPA + FT) group (**d**).

To gain an insight into the function of microbiota, the PICRUSt2 tool [42,49] was used to predict KEGG orthologous genes of the assigned taxa. In total, 5071 KEGG orthologous genes were predicted from all samples (Table S6). The predicted genes belonged to 11 KEGG pathways, and the three most abundant pathways were carbohydrate metabolism, amino acid metabolism, and metabolism of cofactors and vitamins (Figure S8). To determine the enriched or depleted genes within groups, Spearman rank correlation was calculated between the predicted genes within each treatment group and the duration of treatment. The NS control group showed positive correlation with 22 cellular processes and regulation factors along the 33 day period of our study, and riboflavin biosynthesis and proteolysis showed negative correlation (Figure 8a). On the contrary, the PPA group had a positive correlation with porphyrin and biotin metabolism; and a highly negative correlation with acyl-CoA thioesterase (Figure 8b). Other annotations that showed negative association with the duration of PPA treatment were vitamins’ metabolism, ubiquinone biosynthesis, streptomycin biosynthesis, amino and nucleic acids (including histidine) metabolism, and lipid metabolism (Figure 8b). The duration of BF intervention had a positive correlation with vitamin B12 and flagella biosynthesis, and a negative correlation with various cellular processes including succinate biosynthesis, pyruvate metabolism, nitrogen metabolism, beta-Lactam resistance, and peptidoglycan biosynthesis. Within the FT treated group, most of the top orthologs that showed positive correlations with timepoints belonged to flagella biosynthesis. In contrast, arginine and carbohydrate metabolism were among the top orthologs with a negative correlation with the different timepoints (Figure 8d).

## 4. Discussion

ASD is a complex set of neurodevelopmental disorders that are linked with the dysbiosis of gut microbiota and their metabolites [6,22,54,55]. Therefore, modulation of gut microbiota was used as a management strategy for ASD [56]. Here, we evaluated the effect of both the probiotic *Bifidobacterium* and fecal microbiota transplantation treatments on animal behavior and global gut microbiota alteration in propionic acid rat model of autism. Both interventions corrected the social impairment induced by PPA treatment [34], increased the microbial diversity, promoted specific microbiota alterations, and predicted to cause certain manipulation of the microbial metabolic pathways.

Since a growing body of evidence highlights the importance of the gut microbiota-brain axis and its correlation with mental illnesses, such as ASD, several treatment strategies focusing on manipulating the gut microbiota have been developed. This included the utilization of prebiotics, probiotics, and fecal microbiota transplantation [56]. One of the widely used probiotic species is *B. longum*, since it is a major member of gut microbiota with several beneficial effects on human well-being in general and mental health in particular [57,58]. Indeed, *B. longum* improved ASD severity in a number of human and animal experiments [28,59,60]. Fecal microbiota transplantation is another widely used strategy that improved ASD in several studies [61,62,63]. Hence, we utilized these two interventions in propionic acid rat model of autism to determine their effects on animal behavior and gut microbiota modulation.

We evaluated the diversity of rat gut microbiota by sequencing the V3–V4 of 16S rRNA gene. The depth of our sequencing strategy was suitable since similar depth was shown to be adequate to have a comprehensive evaluation of gut microbiota in rats [64]. Moreover, the observed enrichment in gut microbiota of BF and FT treated animals indicates that both treatments caused increased diversity of gut microbiota, which resulted in significant separation of the samples unweighted and weighted UniFrac distances. This observation is in agreement with other reports that utilized FT treatments [25,63]. These findings indicate that each treatment caused significant variation of gut microbiota over the intervention period.

We compared the composition of gut microbiota between and within the treatment groups over the interventions period. We found that PPA treatment resulted in a depletion of Bacteroidia including *Bacteroides* and Bacteroides family S24-7. This finding suggests their possible negative association with ASD. The reduction of *Bacteroides* in animal models of autism and human subjects was observed in number of studies [5,65,66]. On the contrary, the Bacterodales family S24-7, which represents a major proportion of gut microbiota in rodents [67,68,69], showed increased abundance in maternal immune activation (MIA)-induced ASD mouse model, BTBR mouse model of autism, and valproic acid rat model of autism [66,70,71]. The discrepancy between our report and these studies could be attributed to PPA rat models of autism used in our study as opposed to the other used models.

We also observed that gut microbiota of PPA treated animals were enriched with *Streptococcus*, *Paraeggerthella*, and Lachnospiraceae. Few reports investigated the role of *Streptococcus* in ASD. For instance, Finegold et al. demonstrated a higher abundance of *Streptococcus* in healthy children as compared to ASD patients [72]. Moreover, de Angelis et al. highlighted a higher abundance of gut *Streptococcus salivarius* in healthy controls as compared to that of ASD children [16]. In the same report, the relative abundance of *S. thermophilus* in ASD children was higher than that of healthy controls [16], emphasizing a possible contrasting association between different species of streptocci with ASD. However, more studies are needed to investigate their clear role in autism. The association between *Paraeggerthella* and Lachnospiraceae with ASD is documented. For example, *Paraeggerthella* was highly abundant in a *Shank* knock-out mouse model that displayed autism-like phenotypes [73]. Additionally, Lachnospiraceae was found to be increased in ASD children compared to healthy controls [74]. Lachnospiraceae family is composed of about 60 heterogenous genera. Genera within this family are known to dominate gut microbiota including *Dorea*, *Blautia*, *Lachnospira*, *Coprococcus*, *Roseburia*, and *Ruminococcus* [75]. Members of Lachnospiraceae family harbor wide range of metabolic functions that include synthesis of short chain fatty acids (including butyrate), mucin degradation, and sugar and aromatic amino acids metabolism. Their dysbiosis was associated with number of other chronic illnesses such as inflammatory bowel disease, kidney disease, liver diseases, and neurobehavioral diseases [75]. Altogether, PPA treatment resulted in dysbiosis of gut microbiota in rats over 30 days post intervention.

The gut microbiota of BF-treated animals were characterized by early peak in *Bifidobacterium*, accompanied by increased abundance of *Clostridium* in the middle of treatment and a bloom of *Dorea*, the family Lactobacillaceae, and *Marvinbryantia* at the end of treatment. Day 4 represents the day when the animals received *Bifidobacterium* treatment; hence, an increase in *Bifidobacterium* abundance is expected and validates our sequencing approach. *Clostridium* is a predominant member of gut bacteria. Although this genus harbors a number of pathogenic microbes, it contains several other beneficial bacteria that perform essential functions in the gut [76]. For example, *Clostridium* cluster IV and XIVa are groups of bacteria that account for about 35% of intestinal bacteria [77]. *Clostridium* cluster IV is mainly dominated by *Faecalibacterium* and *Anaerofilum* [78,79]. On the other hand, *Clostridium* cluster XIVa is mainly composed by *Eubacterium*, *Roseburia*, *Butyrivibrio*, *Ruminococcus*, *Dorea*, *Coprococcus*, and *Lachnospira* [80]. *Clostridium* cluster IV and XIVa help in the digestion of dietary fibers and complex carbohydrates, which can generate short chain fatty acids (including acetate and butyrate) as metabolic end products. These metabolic end products have several beneficial effects on the host including regulation of gut mucosal immunity and inhibition of inflammation [81,82,83]. Indeed, *Clostridium* cluster IV and XIVa was significantly reduced in autistic patients [84,85], and recently we found a reduced level of *Clostridium* cluster XIVa in a PPA rat model of autism that was corrected with BF and FT treatments [34]. These findings highlight a possible important role of *Clostridium* in autism and the ability of BF treatment to restore their balance. As mentioned earlier, *Dorea* is a major member of *Clostridium* cluster XIVa. On the contrary, Lactobacillaceae is a family of lactic acid producing bacteria, and its members are commonly used as probiotics [86]. In addition, *Marvinbryantia* is a member of Lachnospiraceae that can degrade complex carbohydrates [87]. *Marvinbryantia* was one of the key bacteria to show increased abundance after omega-3 treatment [88], and it was demonstrated that omega-3 improved ASD severity by manipulating gut microbiota [89]. Hence, this could underline a beneficial effect of *Marvinbryantia* in ASD. Together, BF treatment following PPA intervention restored certain members of gut microbiota with possible beneficial impact on mental health.

PPA treatment followed by FT caused reduced abundance of *Akkermansia*, followed by an early peak in the abundance of Bacterodales family S24-7. Then, *Lactonifactor*, Lachnospiraceae, *Butyricicoccus*, *Dorea*, and *Paraeggerthella* were enriched during the two terminal treatment timepoints. *Akkermansia* is a mucolytic organism that is usually enriched in healthy human gut, and their dysbiosis could lead to impaired intestinal barrier [90]. Although *Akkermansia* abundance was significantly decreased in number of ASD studies, other reports showed that it was enriched in patients with ASD [90]. These conflicting findings could be attributed to the different criteria used in ASD diagnosis. Additionally, ASD is a heterogeneous disorder that involves various degrees of severity, which could affect the findings of microbiota reports. Interestingly, we found a negative correlation between the relative abundance of *Akkermansia* with the length of treatment with both FT and BF. This indicates that both treatments results in a gradual reduction of this bacteria in the gut. The peak of Bacterodales family S24-7 abundance during early intervention period is expected since Bacterodales family S24-7 is a dominant member of rodents’ intestine [67,68,69]. Therefore, it is expected to see an increase in their abundance upon receiving fecal microbiota transplantation. *Lactonifactor* is a member of gut bacteria that can produce enterolignans from the digestion of plant Lignans [91], and enterolignans is postulated to have a modulatory effect on the gut-brain axis [92]. As indicated earlier, Lachnospiraceae is a large family of bacteria, and members of this family can be associated with health and disease [75]. For instance, significant group of Lachnospiraceae can be involved in short chain fatty acids synthesis, which has known health promoting properties. On the contrary, their high abundance was positively correlated with number of chronic diseases including type 2 diabetes, obesity, inflammatory bowel disease, and major depressive disorder (reviewed elsewhere [75]). *Dorea* and *Butyricicoccus* are member of *Clostridium* cluster XIVa [93] that did not only show significant increase in the terminal timepoint upon BF and FT treatments, but their abundance was also positively correlated with the days of both treatments. Our findings suggest early variation in microbiota composition. Of note, while our previous publication reported an improvement in autism symptoms by day 33 [34], we cannot rule out an earlier improvement prior to day 33 based on this finding alone. In short, FT treatment resulted in enrichment of certain taxa, and selected members of these taxa were also observed upon BF treatment.

We found certain metabolic pathways with differential abundance in the predicted metagenome of the identified taxa in each treatment group. For instance, PPA-treated animals were predicted to have an increase in gut of porphyrin and biotin metabolism accompanied by a depletion in acyl-CoA thioesterase, vitamins metabolism, ubiquinone biosynthesis, and lipid metabolism. Porphyrins are intermediate metabolite in heme synthesis pathway, and increased levels of urinary porphyrins was observed in ASD children that correlated with autism severity [94,95]. Biotin (vitamin B7) on the other hand is a co-factor that is involved in gluconeogenesis and biosynthesis of fatty acids, and its reduced levels are associated with neuroglial disorders [96]. However, a clear role of porphyrins and biotin in autism is yet to be elucidated. Acyl-CoA thioesterase is an enzyme that is involved in the biosynthesis of fatty acids including butyrate [97,98]. Depletion of acyl-CoA thioesterase in our rat model of autism further highlights the importance of butyrate in maintaining wellbeing in general and mental health in particular. Besides, vitamins deficiency and altered lipid metabolism are known to be associated with neurodevelopmental disorders including ASD [99,100], and ubiquinone supplementation improved the symptoms of ASD in children [101]. Moreover, histidine is an essential amino acid that is involved in number of cellular processes. It can be metabolized to produce histamine, an organic nitrogen compound that is involved in several functions including host immunity and neurotransmission [102]. Accordingly, PPA treatment is predicted to cause deferential abundance of metabolic pathways that are directly or indirectly related to neurodevelopmental disorders.

On the contrary, gut microbiota of BF treated animals are predicted to have higher levels of vitamin B12 biosynthesis, associated with a concomitant decline in succinate biosynthesis, pyruvate and nitrogen metabolism, peptidoglycan biosynthesis and beta-lactam resistance genes. Vitamin B12, or cobalamin, is a cofactor involved in methylation and biosynthesis of antioxidants. Both biological activities were showed to be impaired in ASD. Additionally, B12 supplementation improved the clinical outcomes of the disease [103]. On the contrary, higher levels of succinate biosynthesis and pyruvate and nitrogen metabolism were shown to be associated with ASD [104,105]. However, their exact role in neurodevelopmental disorders is not yet known. The reduced levels of peptidoglycan biosynthesis and beta-lactam resistance genes upon BF treatment indicate that BF supplementation could reduce the level of beta-lactam resistant Gram-positive bacteria. In fact, increased resistance of beta-lactam resistant gene and peptidoglycan biosynthesis were identified as markers of ASD [106,107]. Interestingly, one KEGG orthologous gene in the BF group, and most of the KEGG orthologous genes in FT group, related to flagella biosynthesis were enriched. Flagella is an important factor in bacterial motility and was demonstrated to be enriched in healthy controls compared to autistic children [23]. Higher enrichment of flagella biosynthesis genes in the FT treatment group could be linked to the bloom of Lachnospiraceae, which are known to express heavily flagellated bacteria [108]. Bacterial flagella are known to mediate the antimicrobial C-type lectin through the stimulation of Toll-like receptor 5 (TLR5) [109]. C-type lectin has a great impact on several cellular processes including protection against infection and immune modulation [110]. Indeed, higher level of peripheral blood C-type lectin was observed in mothers with healthy children compared to those with autistic children [111], which underline a potential association between C-type lectin and autism. Since the bacterial flagella biosynthesis was the most enriched pathway upon FT treatment, we could postulate that flagella biosynthesis could have a regulatory effect on gut microbiota through stimulating the expression of C-type lectin. However, this theory needs further investigation. In conclusion, our findings emphasize that BF and FT treatments caused an enrichment of certain bacterial taxa with specific metabolic activity, which may have an impact on mental health.

The main limitation in our study is the inconsistencies between some of our findings and that of published reports. There is no standard model of autism and intervention protocol. Moreover, most studies investigated the impact of various treatments either in human subjects or defined models of autism, and the availability of data demonstrating the dysbiosis of gut microbiota in rat models of autism is limited. Thus, using different animal strains and dosage and duration of probiotics or fecal microbiota transplantation may provide an inaccurate comparison. However, there is significantly high similarity in gut microbiota of rats and mice with that of human, which may justify their use in evaluating the relationship between gut microbiota dysbiosis and various chronic illnesses including ASD. Although PICRUSt provide an accurate prediction of the metabolic properties of the microbiota, metagenomic studies could provide more realistic data about the functional properties of the microbiota. Additionally, there is a lack of complete understanding of the role of predicted metabolic pathways in ASD. Thus, a future fecal metabolomic study with and without the probiotic or fecal microbiota transplantation treatments could provide better understanding of their mechanism in behavioral impairments. Moreover, these mechanistic studies could clarify if the dysbiosis in the microbiota and their metabolite are either a cause or consequences of ASD.

## 5. Conclusions

*B. longum* and fecal microbiota transplantation interventions in PPA-treated rat alter gut microbiota differently. Both treatments reduced the abundance of potentially harmful bacteria and caused an increase of clusters of treatment-specific bacterial taxa. These bacteria are predicted to harbor diverse metabolic pathways in the case of BF treatment, and mainly enriched with genes related to flagella biosynthesis following FT treatment. Further metagenomic and metabolomic studies are needed to better understand the mechanisms involved in the microbiota manipulation associated with each intervention.

## Data Availability

The data presented in this study are available on request from the corresponding author.

## Supplementary Materials

The following supporting information can be downloaded at: https://www.mdpi.com/article/10.3390/nu14030608/s1, Figure S1: Richness (Chao1) and diversity (Shannon) indices of gut microbiota with different treatments, Figure S2: Principal coordinate analysis of unweighted UniFrac distances in each group. Each circle represents one sample, Figure S3: Principal coordinate analysis of unweighted and weighted UniFrac distances at each timepoint, Figure S4: Identified bacterial taxa at the phylum level, Figure S5: Identified genera clustered by sampling day, Figure S6: Linear discriminant analysis (LDA) effect size (LEfSe) of gut microbiota between groups, Figure S7: Associations of the bacterial genera with the timepoints, Figure S8: Predicted Kyoto Encyclopedia of Genes and Genomes (KEGG) pathways, Table S1: Number of sequencing reads per sample, Table S2: Relative abundance of the identified taxa in each sample, Table S3: Relative abundance of the identified phyla in each sample, Table S4: Relative abundance of the identified genera in each sample, Table S5: Spearman correlation between the identified genera and experimental time, Table S6: Relative abundance of the KEGG Orthology annotations in all samples.
