# Supplementary material for: Different Alterations in Gut Microbiota between Bifidobacterium longum and Fecal Microbiota Transplantation Treatments in Propionic Acid Rat Model of Autism
Source: Nutrients. 2022 Jan 30;14(3):608. doi: 10.3390/nu14030608 (PMC8838423; doi:10.3390/nu14030608)
Supplement: Supplementary file 1 [file nutrients-14-00608-s001.zip › Autism_manuscript_012021_Supplementary_data_Correction.pdf]

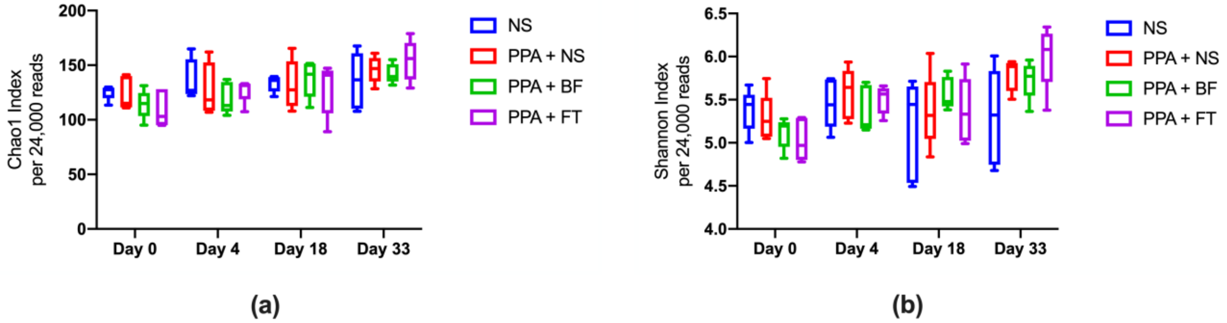

Figure S1. Richness (Chao1) and diversity (Shannon) indices of gut microbiota with different treatments. (a) Chao1, and (b) and Shannon indices of gut microbiota at different treatment was compared at each timepoint. Total number of reads per sample was equalized to 24,000 reads. NS, normal saline group; PPA+NS, propionic acid control group; PPA+BF, *Bifidobacterium* treatment group; PPA+FT, fecal microbiota transplantation treatment.

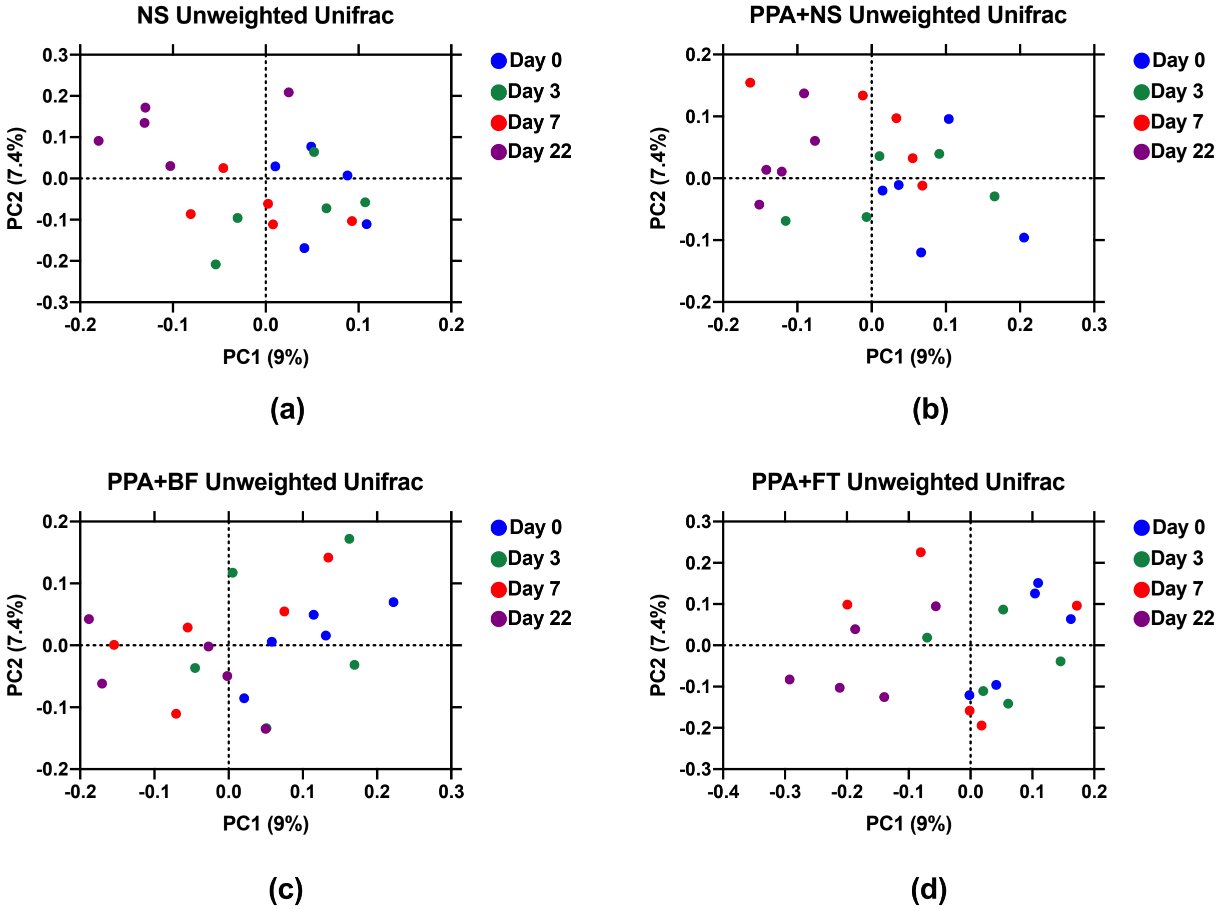

Figure S2. Principal coordinate analysis of unweighted UniFrac distances in each group. Each circle represents one sample. Total number of reads per samples was equalized to 24,000 reads. Normal saline (NS) control group (a), propionic acid (PPA+NS) control group (b), *Bifidobacterium* (PPA+BF) treated group (c), fecal microbiota transplantation treated (PPA+FT) group (d). Percentage of variance explained by each component is presented for each axis.

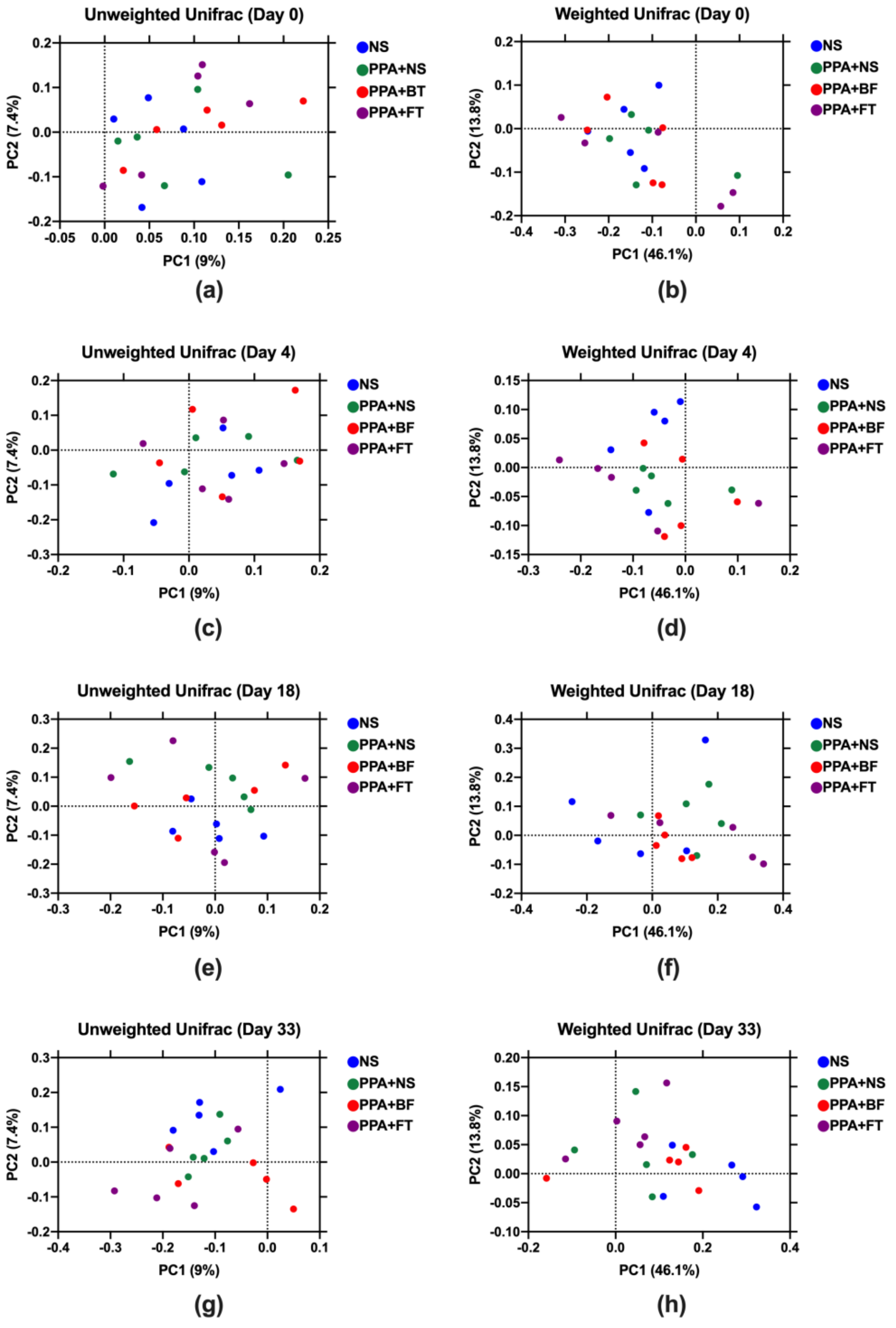

Figure S3. Principal coordinate analysis of unweighted and weighted UniFrac distances at each timepoint. Each circle represents one sample. Left panels represent unweighted UniFrac distances and right panels represent weighted UniFrac distances. Samples are clustered at day 0 (a and b), day 4 (c and d), day 18 (e and f), and day 33 (g and h). Percentage of variance explained by each component is presented for each axis. NS, normal saline; PPA, propionic acid; BF, *Bifidobacterium*; FT, fecal microbiota transplantation.

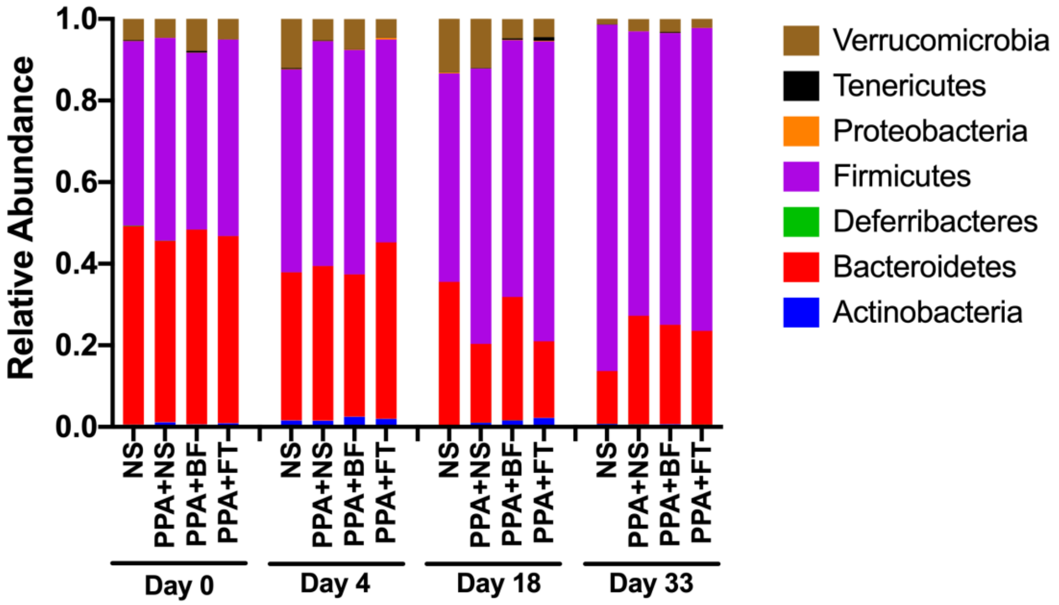

Figure S4. Identified bacterial taxa at the phylum level. Relative abundance of assigned bacterial phyla in all samples are clustered at each timepoint. Each column represents the mean relative abundance of the identified phyla at each timepoint per group.

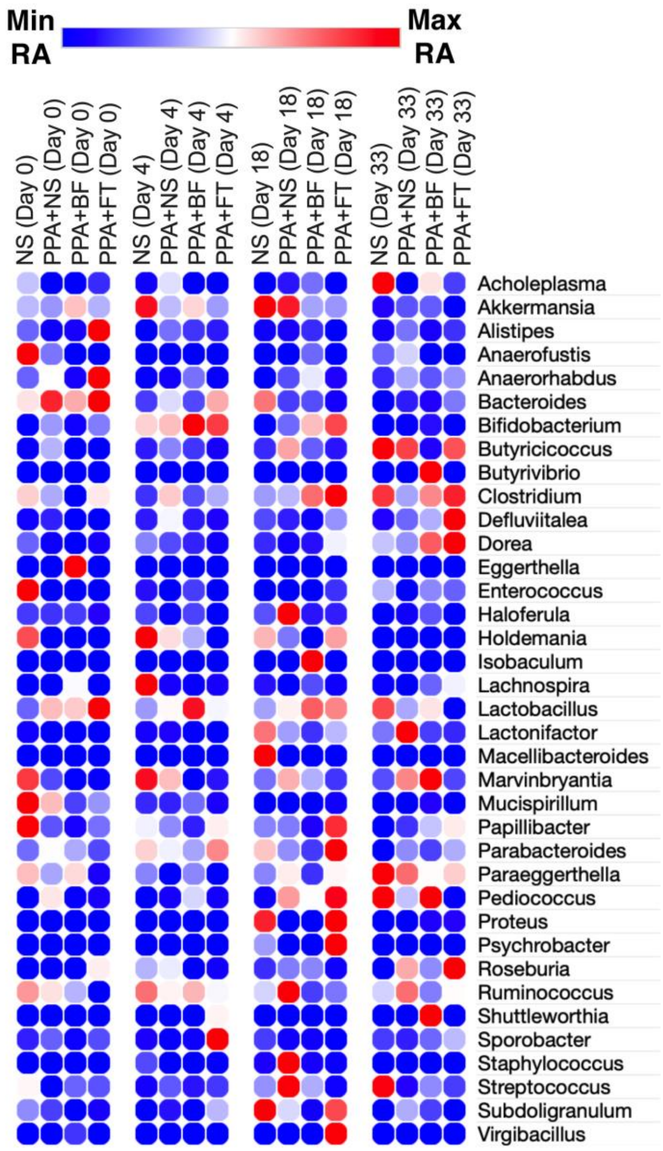

Figure S5. Identified genera clustered by sampling day. Each circle represents the mean relative abundance of the identified genus from animals within a treatment group at each timepoint (*n* = 5). NS, Normal saline control group; PPA+NS; propionic acid control group; PPA+BF, *Bifidobacterium* treated group; PPA+FT, fecal microbiota transplantation treated group. Scale represents a color scheme for the minimum and maximum relative abundance (RA) values in each row.

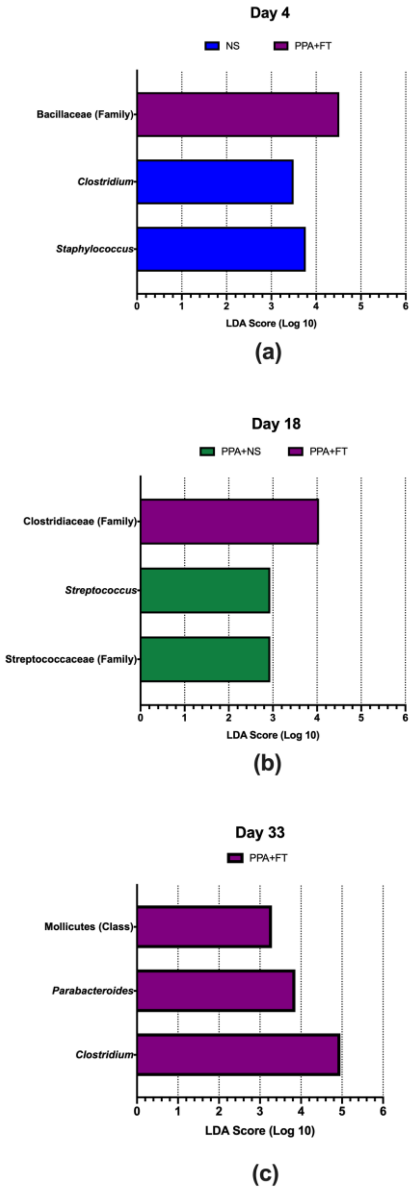

Figure S6. Linear discriminant analysis (LDA) effect size (LEfSe) of gut microbiota between groups. Relative abundance of gut microbiota was compared between the different treatment groups at day 4 (a), day 18 (b) and day 33 (c). Only taxa with statistically significant differences ($p \leq 0.05$) are presented. NS, Normal saline control group; PPA+NS; propionic acid control group; PPA+FT, fecal microbiota transplantation treated group.

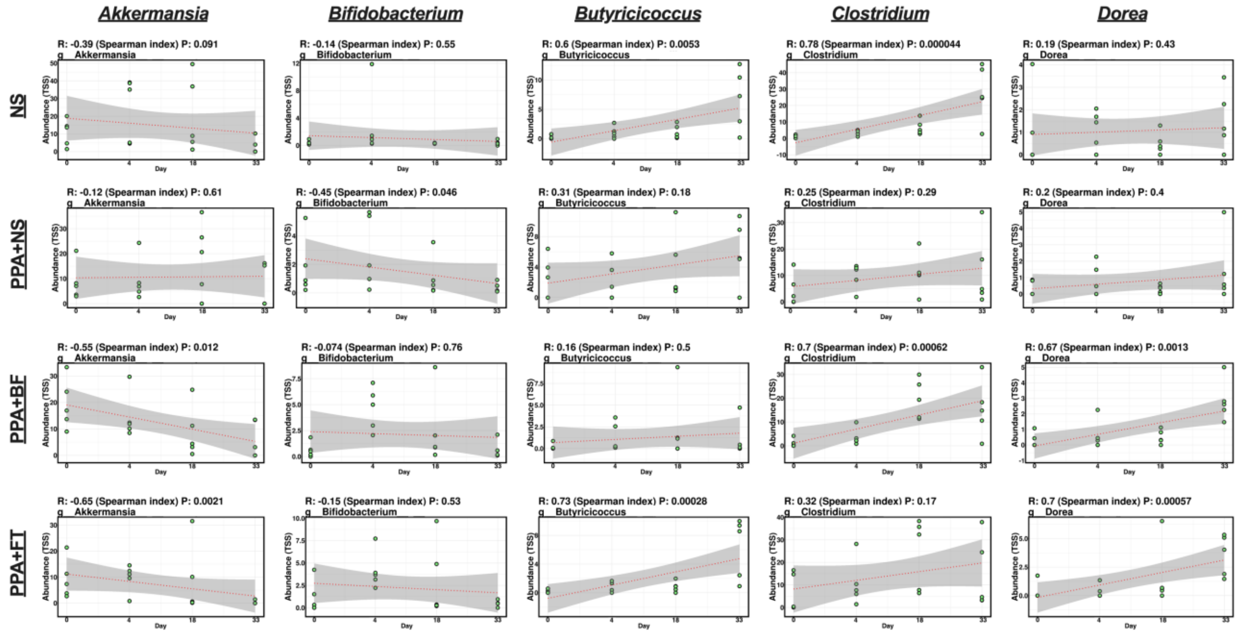

Figure S7. Associations of the bacterial genera with the timepoints. Spearman rank correlation coefficient (R) was calculated between the identified genera and experimental time, and top 5 genera with the highest correlation are presented. Regression lines are depicted in red, and shaded regions denote 95% confidence intervals. TSS, total sum scaling normalization of abundance; NS, Normal saline control group; PPA+NS; propionic acid control group; PPA+BF, *Bifidobacterium* treated group; PPA+FT, fecal microbiota transplantation treated group.

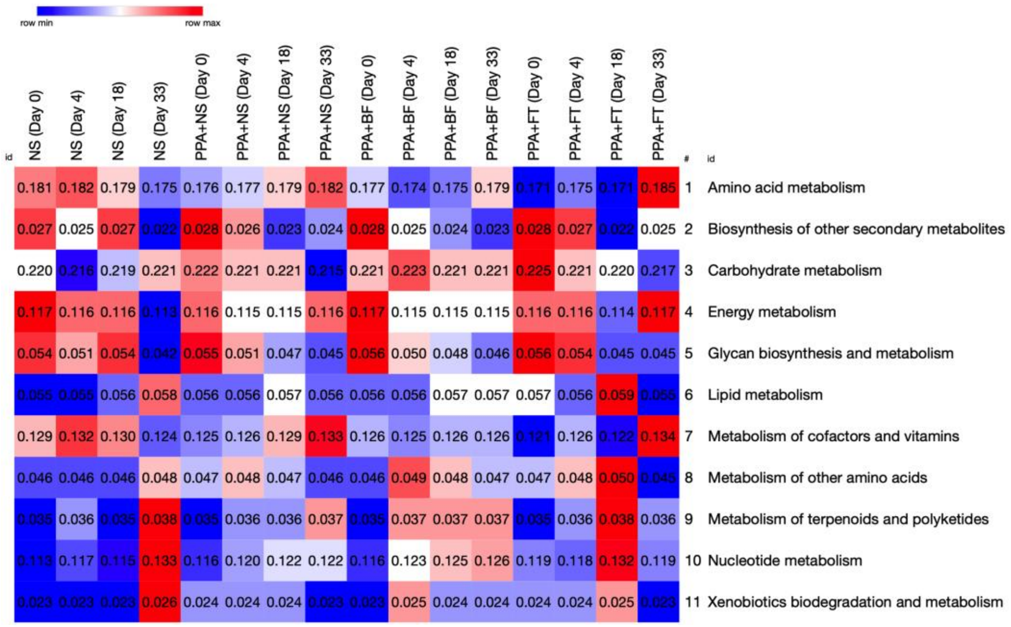

Figure S8. Predicted Kyoto Encyclopedia of Genes and Genomes (KEGG) pathways. Relative abundance of predicted pathways is presented. Each square represents the mean relative abundance of the predicted pathway from 5 animals in each group at the designated timepoint. Scale represents a color scheme for the minimum and maximum relative abundance values in each row. Heatmap was generated using Morpheus (https://software.broadinstitute.org/morpheus).
